# Reversing diet-induced metabolic dysregulation by diet switching leads to altered hepatic *de novo* lipogenesis and glycerolipid synthesis

**DOI:** 10.1038/srep27541

**Published:** 2016-06-07

**Authors:** Greg M. Kowalski, Steven Hamley, Ahrathy Selathurai, Joachim Kloehn, David P. De Souza, Sean O’Callaghan, Brunda Nijagal, Dedreia L. Tull, Malcolm J. McConville, Clinton R. Bruce

**Affiliations:** 1 Centre for Physical Activity and Nutrition Research, School of Exercise and Nutrition Sciences, Deakin University, Burwood, Australia; 2 Metabolomics Australia, Bio21 Molecular Science and Biotechnology Institute, Parkville, Australia

## Abstract

In humans, low-energy diets rapidly reduce hepatic fat and improve/normalise glycemic control. Due to difficulties in obtaining human liver, little is known about changes to the lipid species and pathway fluxes that occur under these conditions. Using a combination of stable isotope, and targeted metabolomic approaches we investigated the acute (7–9 days) hepatic effects of switching high-fat high-sucrose diet (HFD) fed obese mice back to a chow diet. Upon the switch, energy intake was reduced, resulting in reductions of fat mass and hepatic triacyl- and diacylglycerol. However, these parameters were still elevated compared to chow fed mice, thus representing an intermediate phenotype. Nonetheless, glucose intolerance and hyperinsulinemia were completely normalized. The diet reversal resulted in marked reductions in hepatic *de novo* lipogenesis when compared to the chow and HFD groups. Compared with HFD, glycerolipid synthesis was reduced in the reversal animals, however it remained elevated above that of chow controls, indicating that despite experiencing a net loss in lipid stores, the liver was still actively esterifying available fatty acids at rates higher than that in chow control mice. This effect likely promotes the re-esterification of excess free fatty acids released from the breakdown of adipose depots during the weight loss period.

Type 2 Diabetes (T2D) represents a global health crisis^1^. Understanding the mechanisms responsible for defective glucose homeostasis is of fundamental importance. The approach to elucidating mechanisms has relied on a forward thinking method of problem solving, involving examination of pathogenic mechanisms during the process of disease development. This has revealed that defects in liver glucose metabolism are an early feature of deteriorating metabolic health^2^. Indeed, studies in humans^3^,^4^,^5^, dogs^6^,^7^,^8^,^9^, and rodents^10^,^11^,^12^,^13^,^14^,^15^ have shown that a deterioration in hepatic insulin and/or glucose action (i.e. glucose effectiveness) occurs rapidly in response to nutrient oversupply, and thus represents a primary pathogenic manifestation. While the exact mechanisms underlying the impairment of hepatic glucose metabolism are not entirely clear, excess hepatic lipid accumulation appears to be important^14^,^16^. Indeed, glucose intolerance and liver insulin resistance are associated with increased hepatic triacylglycerol (TAG) and diacylglycerol (DAG)^14^,^16^. However, there are several instances where alterations in hepatic lipid accumulation and glucose homeostasis do not correlate^17^,^18^,^19^. Thus, there is some controversy regarding the role of hepatic lipids in regulating insulin action and glucose metabolism.

A way to address this is to examine how liver lipids are altered following reversal of glucose intolerance and insulin resistance. In humans with T2D, it is clear that glycemic control can be rapidly normalized by either bariatric surgery^20^,^21^ or very-low energy diets^22^,^23^,^24^,^25^,^26^ with much of this being liver mediated^20^,^22^,^23^,^24^,^26^. The underlying mechanisms responsible, however, are unclear. While the improvement in liver glucose metabolism in response to energy restriction is associated with reduced hepatic TAG^23^, little is known about effects on bioactive lipids such as DAG, due to the difficulty in obtaining human liver samples. Our previous studies in mice^14^ demonstrated that hepatic insulin resistance is the earliest metabolic manifestation of a high-fat high-sucrose diet (HFD), which was associated with ectopic lipid accumulation, but not hepatic or adipose tissue inflammation. In light of these studies, we aimed to conduct a comprehensive analysis of lipid and glucose metabolism in mice that have undergone an acute switch from a HFD back to a standard chow diet in order to better define the relationship between hepatic lipid metabolism and glucose homeostasis.

## Experimental Procedures

### Study design

Four week old male C57Bl/6 mice (N = 60; Animal Resources Centre, Australia), were maintained at 22 ± 1 °C on a 12/12 h light/dark cycle, with free access to water and a standard chow diet (9% energy from fat, Barastoc Rat & Mouse, Ridley AgriProducts, Australia). Mice were housed 5 per cage and acclimatised to the facility for 4 weeks. At 8 weeks of age, mice were randomly allocated to one of the following groups: 1) continuation on a chow diet (CHOW; N = 20) for 65 days; 2) HFD (N = 20; 42% energy from fat and 20% from sucrose; Specialty Feeds SF4-001, Australia) for 65 days; or 3) HFD for 56 days followed by 9 days of *ad libitum* chow (HFD → CHOW; N = 20). Over the initial 56 days, body mass was measured weekly while food intake was measured daily for 5 days per week. Between day 57–65 (the diet switch period), body mass and food intake were measured daily. On day 42 a blood sample was obtained after a 5 h fast (food removed at 07:00 h) to measure glucose, plasma insulin and free fatty acids (FFA). An oral glucose tolerance test (OGTT) was then performed (described below) to verify diet-induced glucose intolerance. On day 63 (7 days post-diet switch) a stable isotope labelled OGTT was conducted following a 5 h fast (described below). On day 64, ^2^H_2_O was administered to determine hepatic glucose and lipid fluxes. On day 65 mice were humanely killed and the liver was immediately freeze clamped. Other tissues (quadriceps, epididymal and subcutaneous fat) were collected and weighed. Experiments were approved by the Deakin University Animal Ethics Committee and performed according to the *Guide for the care and use of laboratory animals*, Eighth edition (2011).

### OGTT

On day 42, following a 5 h fast, blood (~30 μL) was obtained from the tail vein for determination of glucose, plasma insulin and FFA. Glucose (50 mg) was administered via oral gavage and blood obtained at 15, 30, 45, 60, 90 and 120 min. On day 63 a stable isotope labelled OGTT was performed^27^. The administration of isotopically labelled glucose provides assessment of dynamic glucose disposal, pattern of endogenous glucose production (EGP) and hepatic futile glucose cycling^27^. Glucose was measured with a glucose meter (Accu-Check, Roche, NSW, Australia) and plasma tracer enrichment measured by gas chromatography-mass spectrometry (GC–MS)^27^.

### Determination of the sources of EGP

At 17:00 h on day 64, mice received an intraperitoneal injection of ^2^H_2_O (30 mL/kg, Sigma, Castle Hill, NSW, Australia) containing 0.9% (w/v) NaCl. Mice were maintained on drinking water enriched with 10% ^2^H_2_O overnight which achieved body water enrichment of ~5%. The next morning blood (~50 μL) was obtained after a 5 h fast for determination of ^2^H body water labelling as well as positional enrichment of ^2^H in plasma glucose by GC-MS which allows the proportion of glucose produced from gluconeogenesis (GNG) and glycogenolysis to be resolved^27^,^28^.

### Lipid metabolism

TAG was determined using a colorimetric assay (Triglycerides GPOPAP; Roche Diagnostics, Indianapolis, IN). DAG and ceramide were extracted from ~15 mg of liver as described in^29^ and analyzed as detailed in the LC-MS/MS method in Weir *et al.*^30^. Glycerolipid synthesis and *de novo* lipogenesis (DNL) were measured using ^2^H_2_O labelling. Specifically, glycerolipid synthesis was determined by measuring ^2^H incorporation into the glyceride glycerol moiety of the hepatic glycerolipid pool^31^ while DNL was measured by determining the incorporation of ^2^H into total palmitate^32^,^33^. Palmitate labelling is used for DNL measurements as it is the major product of the fatty acid synthase reaction in mammals, with labelling in fatty acids that are >16 carbons in length resulting from chain elongation, not DNL^33^,^34^. Lipid extraction on ~15 mg of pre-weighed liver tissue was performed according to the methods of Folch^35^ with the addition of [U-^13^C] palmitate (Cambridge Isotopes) as an internal standard which enabled quantification of total palmitate^35^. The lipid fraction was transesterified by addition of 3 N methanolic HCl (Sigma, Castle Hill, Australia) and was incubated at 60 °C for 1 h. Glycerol was separated from the fatty acid methyl esters (FAME) by Folch extraction^35^. The aqueous phase containing glycerol was dried and derivatized by addition of MTBSTFA + 1% t-BDMCS (Sigma-Aldrich) and samples were analyzed using electron ionization GC-MS. Specifically, the molecular [M-57] ions of *m/z* 377 and 378 were analysed in SIM mode. The FAME containing lipid fraction was dried and resuspended in hexane. The molecular ions of *m/z* 270, 271 and 286 ([U-^13^C] palmitate internal standard) of methylpalmitate were analyzed in SIM mode via GC-MS. Glycerolipid synthesis and DNL were calculated according to the precursor product labelling ratio equation:


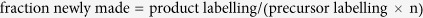


Product labelling refers to the excess molar enrichment of ^2^H in glycerol or palmitate, precursor labelling = body water ^2^H enrichment (plasma) and *n* *=* the number of exchangeable hydrogen atoms, which has been determined to be ~4.5 for hepatic glyceride glycerol^31^ and ~22 for liver palmitate^32^,^33^. Absolute amounts of product synthesized were calculated by multiplying the fraction of newly made product by the absolute product concentration. Correction for background isotopic abundance was performed by subtracting the enrichment of ^2^H_2_O treated samples from that of mouse liver samples not exposed to ^2^H_2_O.

### Liver metabolites

Metabolites were extracted and analysed by GC-MS^36^,^37^. Glycogen was measured in KOH digests subjected to enzymatic hydrolysis followed by quantification of free glucosyl units via glucose oxidase assay.

### Plasma analysis

Plasma insulin and leptin were measured by ELISA (Millipore, St Louis, MO, USA). Plasma FFAs were measured spectrophotometrically by an enzymatic colorimetric assay (NEFA C Kit; Wako Chemicals, Richmond, VA, USA).

### Statistics

Data are presented as mean ± SEM. Data were analysed by one-way ANOVA or two-way repeated measures ANOVA where appropriate. For one-way ANOVA, Newman-Keuls multiple comparisons tests were used to establish differences between groups. For two-way repeated measures ANOVA, Holm-Sidak’s multiple comparisons tests were used to determine differences between groups. Statistical significance was set at p < 0.05.

## Results

### Energy intake and body mass

**Figure 1:**
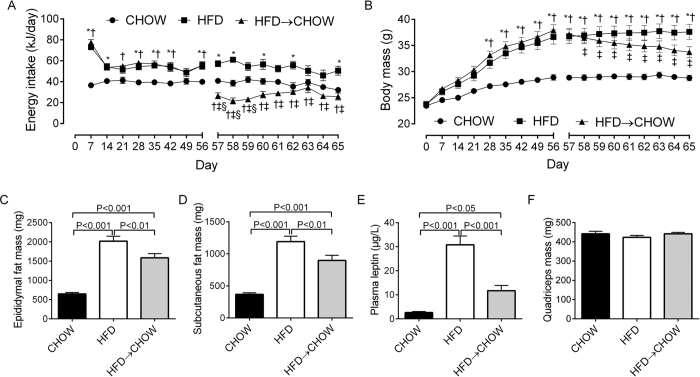
Effects of diet on energy intake and body mass. Energy intake (**A**), body mass (**B**), epididymal (**C**) and subcutaneous fat pad mass (**D**), plasma leptin levels (**E**) and quadriceps muscle mass (**F**). Data are mean ± SEM. N = 20 per group. *HFD vs. CHOW, P < 0.05; ^†^HFD → CHOW vs. CHOW, P < 0.05; ^‡^HFD → CHOW significantly different to day 56, P < 0.05; ^§^HFD vs. HFD → CHOW, P < 0.05.

Over the initial 56 days, the HFD and HFD → CHOW groups consumed more energy than the CHOW group (Fig. 1A). From day 57 to 65 (i.e. the diet reversal), energy intake was reduced in HFD → CHOW (Fig. 1A). Body mass was not different at baseline between the groups (Fig. 1B). However, both the HFD and HFD → CHOW groups gained more body mass than CHOW mice over the 56 day HFD period. At day 65, 9 days after the diet reversal, HFD → CHOW mice lost weight but were not significantly lighter than the HFD mice, and remained heavier than the CHOW group (Fig. 1B). The epididymal and subcutaneous (Fig. 1C,D) fat pads were heavier in HFD compared to the CHOW and HFD → CHOW mice, while fat pad mass remained elevated in HFD → CHOW compared to the CHOW group (Fig. 1C,D). Plasma leptin followed a similar pattern (Fig. 1E). There were no differences in quadriceps mass between groups (Fig. 1F), indicating that weight loss in the HFD → CHOW mice was attributed to loss of fat, not muscle mass.

### Glucose metabolism

**Figure 2:**
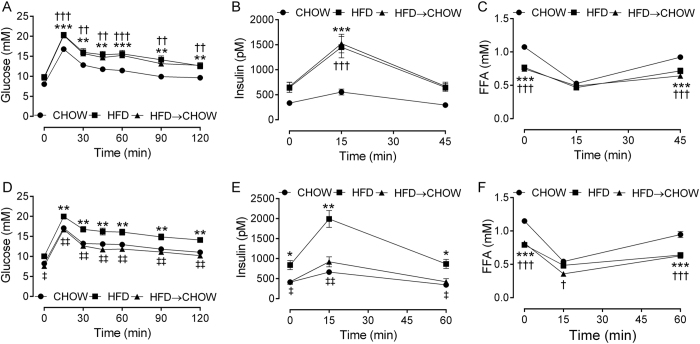
Effects of diet on glucose tolerance. Blood glucose (**A**), plasma insulin (**B**) and FFAs (**C**) during the OGTT performed 14 days prior to diet switch. Blood glucose (**D**), plasma insulin (**E**) and FFAs (**F**) during the stable isotope labelled OGTT performed 7 days after diet switch. Data are mean ± SEM. N = 20 per group. *HFD vs. CHOW, P < 0.05; **HFD vs. CHOW, P < 0.01; ***HFD vs. CHOW, P < 0.001; ^†^HFD → CHOW vs. CHOW, P < 0.05; ^††^HFD → CHOW vs. CHOW, P < 0.01; ^†††^HFD → CHOW vs. CHOW, P < 0.001; ^‡^HFD vs. HFD → CHOW, P < 0.05; ^‡‡^HFD vs. HFD → CHOW, P < 0.01.

**Figure 3:**
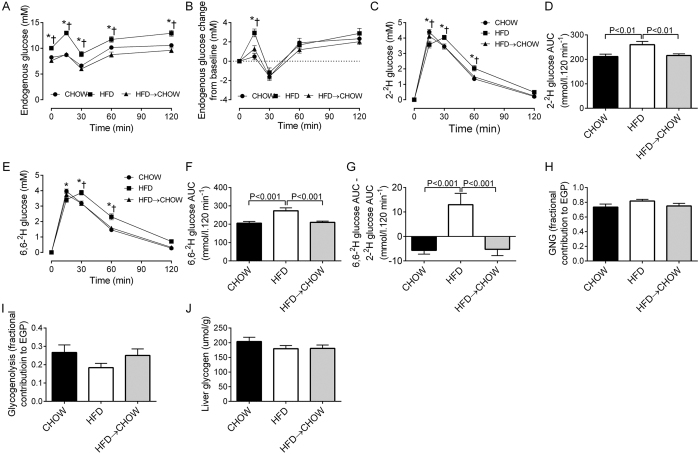
Effects of diet on endogenous glucose, glucose disposal and sources of EGP. Endogenous glucose levels (**A**), the pattern of endogenous glucose production (**B**), disposal of 2-^2^H glucose (**C,D**) and 6,6-^2^H glucose (**E,F**) and hepatic futile glucose cycling (**G**) during the stable isotope labelled OGTT. The fractional contribution of GNG (**H**), and glycogenolysis (**I**) to EGP and liver glycogen (**J**) following a 5 h fast. Data are mean ± SEM. N = 20 per group. *HFD vs. CHOW, P < 0.05; ^†^HFD → CHOW vs. CHOW, P < 0.05.

Prior to diet reversal, the HFD and HFD → CHOW groups exhibited the same degree of glucose intolerance and hyperinsulinemia compared with the CHOW group (Fig. 2A,B). Furthermore, fasting plasma FFAs were lower in the HFD and HFD → CHOW animals (Fig. 2C), but were suppressed to similar levels as the CHOW mice during the OGTT. Importantly, the glucose, insulin and FFA responses were identical between the HFD and HFD → CHOW groups (Fig. 2A–C). After 7 days of switching to a chow diet, glucose tolerance and hyperinsulinemia in the HFD → CHOW group was completely normalized to that of CHOW mice (Fig. 2D,E). In contrast, the plasma FFA profile of the HFD → CHOW mice was unaffected and resembled that of the HFD group (Fig. 2F). Using a stable isotope labelled OGTT, we resolved the blood glucose concentration into the fraction derived from endogenous sources or from the oral glucose load (Fig. 3A–G). Although endogenous glucose was higher in the HFD mice compared with both the CHOW and HFD → CHOW mice, the change from baseline was similar in all groups, except for the 15 min time point where it was higher in the HFD group (Fig. 3B). Load derived glucose was higher in the HFD mice, particularly between 15 to 60 min, and was accompanied by an increase in the 2-^2^H and 6,6-^2^H glucose area under the curve (AUC) when compared with the CHOW and HFD → CHOW mice indicting a defect in glucose disposal (Fig. 3C–F). The improvement in glucose tolerance following the diet switch was due to enhanced glucose disposal such that the concentrations of load derived glucose in the HFD → CHOW mice was restored to those in the CHOW group (Fig. 3C–F). Hepatic futile glucose cycling, which provides an index of the efficiency of hepatic glucose uptake by quantifying the activities of hepatic glucokinase relative to that of glucose-6-phosphatase, as determined by the difference in the 6,6-^2^H and 2-^2^H glucose AUC (Fig. 3G)^27^, was higher in the HFD group compared with the other groups, while this was completely normalised in HFD → CHOW mice (Fig. 3G). Given the rapid normalisation of hyperglycemia and hyperinsulinemia in the HFD → CHOW group, we examined whether the contribution of GNG and glycogenolysis to EGP would be altered. Neither GNG nor glycogenolysis were changed in either the HFD or HFD → CHOW groups, (Fig. 3H,I), nor was liver glycogen content affected (Fig. 3J).

### Liver metabolites

**Figure 4:**
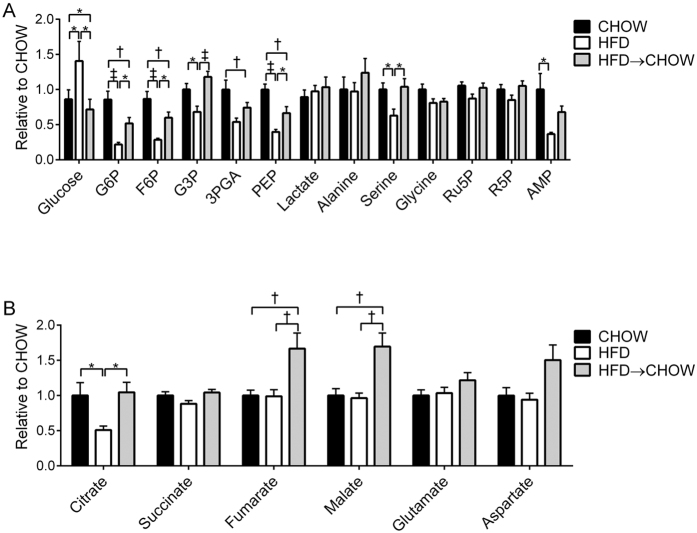
Metabolomic profiling of liver. Glycolytic (**A**) and TCA cycle (**B**) metabolites. Data are mean ± SEM. N = 10 per group *P < 0.05; ^†^P < 0.01; ^‡^P < 0.001 for specified comparisons.

Targeted profiling was performed to analyse metabolites in the glycolytic/GNG pathway as well as the TCA cycle (Fig. 4A). The glycolytic metabolites glucose-6-phosphate (G6P), fructose-6-phosphate (F6P), glycerol-3-phosphate (G3P) and phosphenolpyruvate (PEP), together with the glycolytically derived amino acid serine were all reduced in HFD mice (Fig. 4A). The HFD caused a reduction in adenosine monophosphate (AMP; Fig. 4B), indicating an elevated hepatic energy state. These metabolites were either partially (G6P, F6P, PEP and AMP) or fully restored (G3P and serine) to the levels found in the livers of CHOW mice after switching from a HFD back to chow (Fig. 4A). No differences were found in glycine, ribulose-5-phosphate (Ru5P), ribose-5-phosphate (R5P), lactate or alanine (Fig. 4A). Citrate was reduced in the HFD group compared with both the CHOW and HFD → CHOW groups (Fig. 4B), whereas fumarate and malate were elevated in the HFD → CHOW mice compared with the CHOW and HFD groups (Fig. 4B). Succinate, glutamate and aspartate were not different between groups (Fig. 4B). As expected, hepatic free glucose concentration (Fig. 4A) reflected that of the fasting glucose concentration (Fig. 2D) such that it was elevated in HFD mice and was normalized to that of the CHOW group following dietary switch.

### Hepatic lipid metabolism

**Figure 5:**
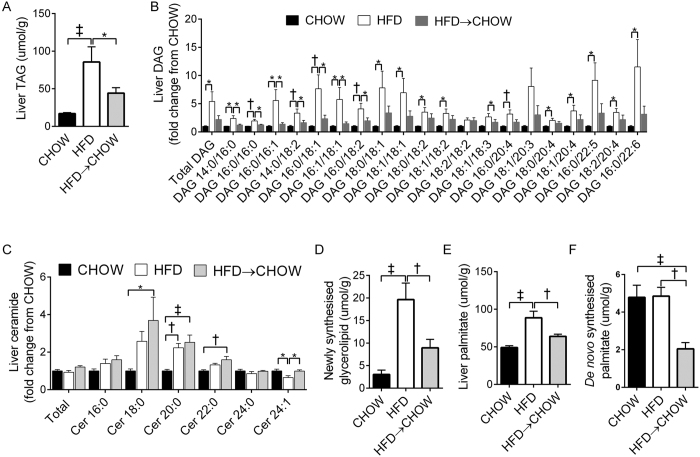
Effects of diet on hepatic lipid metabolism. Liver TAG (**A**), DAG (**B**) and ceramide (**C**) levels as well as the newly synthesized glycerolipid (**D**), total palmitate concentration (**E**) and *de novo* synthesized palmitate (**F**). Data are mean ± SEM. Figure A. N = 20, Figs B-F N = 10/group. *P < 0.05; ^†^P < 0.01; ^‡^P < 0.001 for specified comparisons. TAG, triacylglycerol; DAG, diacylglycerol; Cer, ceramide.

The HFD caused an increase in liver TAG and DAG (Fig. 5A,B). Switching to a chow diet reduced TAG and DAG in the HFD → CHOW mice such that they were not statistically different to those in the CHOW group (Fig. 5A,B). However, while not statistically different, TAG and DAG were still ~2-fold higher in the diet reversal group compared to the CHOW (Fig. 5A,B). In addition, a number of DAG species were increased in the HFD mice (Fig. 4B), while there was a strong trend for these to be reduced with the switch to a chow diet. Total ceramide was not altered by diet (Fig. 5C), but effects on individual species were found such that the HFD increased ceramide 20:0 whereas ceramide 24:1 was reduced (Fig. 5C). Interestingly, ceramide 18:0, 20:0 and 22:0 were elevated in the HFD → CHOW group when compared with the CHOW mice (Fig. 5C). To examine mechanisms responsible for the reduction in liver lipids in the HFD → CHOW mice, we assessed hepatic glycerolipid synthesis and DNL using ^2^H_2_O labelling. The absolute amount of newly synthesized glycerolipid was higher in the HFD group when compared with the HFD → CHOW and CHOW groups (Fig. 5D). Total liver palmitate levels were elevated in the HFD mice, while switching to a CHOW partly normalised hepatic palmitate content (Fig. 5E). The absolute amount of newly synthesized palmitate (DNL) was not different between the CHOW and HFD groups (Fig. 5F). However, DNL was lower in the HFD → CHOW mice compared with those in the CHOW and HFD groups (Fig. 5F).

## Discussion

We show that a rapid reduction in voluntary energy intake, as a result of HFD mice being switched back to a chow diet, completely normalizes glucose intolerance, fasting hyperglycemia and hyperinsulinemia after only 7 days. Interestingly, as in humans^20^,^22^,^23^,^24^,^26^, this occurred even though there was only a modest reduction in body weight and fat pad mass. Moreover, despite the rapid improvement in glucose homeostasis in the diet reversal mice, liver lipids were not completely normalised and remained ~2-fold higher than those in the CHOW group. Together, these findings suggest that increased adiposity and elevated hepatic lipids are not necessarily involved in maintaining the insulin resistant and glucose intolerant state, and that acute changes in energy balance are likely to play a more important role in modulating glucose homeostasis.

Our data clearly demonstrate that HFD-induced defects in glucose tolerance can be completely and rapidly normalized by switching to a chow diet. To inform on the mechanisms, we used a stable isotope labelled OGTT allowing blood glucose to be resolved into that derived from endogenous sources or from the oral glucose load. While the pattern of endogenous glucose concentrations throughout the OGTT were relatively similar between all groups, the disposal of the oral glucose load was impaired in the HFD mice as evident by a greater AUC for both glucose tracers. This defect in glucose disposal was completely normalised with the diet reversal. Our findings suggest that this impairment in glucose disposal in the HFD mice is, at least partially, mediated by an impairment in hepatic glucose uptake. This is supported by the fact that HFD mice exhibited increased hepatic futile glucose cycling indicating a lower hepatic glucose phosphorylation potential. Consistent with this, the HFD caused a reduction in hepatic G6P and other intermediates in the glycolytic/GNG pathway including F6P, G3P, 3-phosphoglycerate (3PGA) and PEP. This is similar to studies in dogs which have consistently shown that a HFD or overfeeding impairs hepatic glucose uptake^6^,^7^,^8^,^38^. Hepatic futile glucose cycling was normalised in the diet reversal group and was associated with a partial restoration of glycolytic/GNG intermediates. In contrast, the only change found in the TCA cycle with the HFD was a reduction in citrate, which upon diet reversal returned to levels observed in the CHOW group. Thus, the changes that occur in hepatic glucose uptake and central carbon metabolism in response to a HFD, can be largely normalized by dietary reversal, indicating that liver metabolism is exquisitely sensitive to changes in energy intake and macronutrient composition.

We also used ^2^H_2_O labelling^39^ to examine the contribution of GNG and glycogenolysis to EGP. Although the HFD mice exhibited fasting hyperglycemia and glucose intolerance, the reliance on GNG and glycogenolysis was not altered. Moreover, these fluxes were not affected in the diet reversal mice. While these results may appear to be unexpected, an elevation in fractional or absolute rates of GNG are only seen in severely hyperglycemic patients with insulin deficiency, for review see^39^. Therefore, it is not surprising that GNG was not elevated in the HFD mice as they are not overtly T2D and insulinopenic, but rather this is a model of pre-diabetes characterised by insulin resistance and hyperinsulinemia^2^. Consistent with our previous findings^27^ and that of others^40^, we show that GNG accounts for ~80% of EGP in acutely fasted mice, reflecting the extremely high metabolic rate of these animals^2^. Thus, contrary to the widely held belief, the reliance on GNG is not increased in HFD mice, and given that we^14^ and others^41^ have shown that basal EGP is not elevated in fat-fed rodents, absolute rates of GNG would not be increased with a HFD.

Previous studies in mice have also shown either complete or near complete normalization of glucose homeostasis in HFD mice when switched back to an *ad libitum* chow diet^42^,^43^,^44^. However, it is important to highlight that these studies used longer term dietary reversal periods of between two weeks to four months^42^,^43^,^44^,^45^. Interestingly, only one study performed any organ specific biochemical measurements^43^. Li *et al.*^43^ found that in mice fed a HFD for 20 weeks, three weeks of CHOW feeding resulted in an improvement in glucose tolerance and caused an almost complete normalization of hepatic TAG and DAG. In contrast, we found that hepatic TAG and DAG were reduced by ~50% with the switch from the HFD to CHOW. Although the TAG and DAG content was not statistically different between the CHOW and diet reversal groups, their levels remained ~2-fold higher. The discrepancies between studies are likely to be attributed to the fact that the dietary reversal period used in the current study was shorter than that employed by Li *et al.*^43^ (i.e. 9 days vs. 3 weeks). Nonetheless, despite the fact that hepatic TAG and DAG were only partially normalised with the diet reversal, there was a complete recovery of glucose homeostasis. This suggests that the relationship between hepatic glycerolipids and glucose homeostasis is complex, and rather than being linear there may be a threshold at which the higher levels of TAG and DAG impair systemic glucose metabolism.

Consistent with our previous findings^14^,^27^, we did not observe any change in total hepatic ceramide content with the HFD. Interestingly, upon analysis of the individual species it became evident that that the HFD caused an increase in ceramide 20:0, whereas ceramide 24:1 was reduced. To our surprise, ceramide 18:0, 20:0 and 22:0 were all elevated in the diet reversal mice when compared with the CHOW group. Thus, unlike the uniform changes seen with hepatic TAG and DAG, we find little relationship between hepatic ceramide and glucose homeostasis, adiposity or hepatic steatosis, which is consistent with the findings of others^46^.

Although it is generally accepted that obese humans have an elevation in plasma FFAs, it is important to point out that this is not always the case, as reviewed by Karpe *et al.*^47^. Consistent with our previous work^27^,^48^ and that of others in mice^49^ and rats^41^,^50^,^51^,^52^, we found that the HFD fed obese mice had consistently *lower* plasma FFA levels than the lean chow controls. A likely explanation for this is that animals fed a HFD have an enhanced efficiency in tissue fatty acid uptake^50^. In addition, as we have shown here, the liver of HFD fed mice also exhibits a dramatic increase in glycerolipid synthesis rates which indicates that plasma FFAs and are being rapidly esterified and stored, thus reducing their concentration.

An unexpected finding was that the plasma FFA concentrations in the diet reversal mice were identical to that of the HFD group (i.e. lower than CHOW). The reason for this did not become apparent until hepatic lipid fluxes were determined. Glycerolipid synthesis was markedly elevated in the HFD mice when compared to both CHOW and reversal groups, explaining the increase in TAG and DAG in these animals and indicating high rates of fatty acid esterification. Despite the dietary reversal mice having a dramatic, but not complete reduction of liver TAG and DAG levels, glycerolipid synthesis, while not reaching statistical significance by one-way ANOVA, was mathematically increased (~2-fold elevation) when compared with the CHOW mice. This was unexpected given energy intake in the diet reversal group was lower than the CHOW mice. In contrast, hepatic DNL was reduced with the diet reversal, while no differences were observed between the CHOW and HFD groups. These data demonstrate that HFD-induced hepatic steatosis in mice is largely caused by excess esterification of dietary fatty acids and not those derived from DNL, which is consistent with other findings in rodents^34^,^53^,^54^. However, upon dietary reversal, the resultant reduction in energy intake reduced hepatic DNL, most likely due to its high energy cost. We propose that the elevated rates of glycerolipid synthesis allow for greater re-esterification of plasma FFAs, likely explaining why the diet reversal mice maintain the same plasma FFA profile as the HFD group. Given the rapid loss of fat mass in these mice, indicating net lipolytic flux from adipose tissue, maintaining elevated rates of hepatic glycerolipid synthesis could prevent increases in plasma FFAs which could be cytotoxic. Thus, DNL is sensitive to a reduction in energy intake, such that when DNL is reduced the liver can maintain relatively high rates of lipid re-esterification to effectively ‘soak up’ the FFAs from the rapidly diminishing adipose depots. However, hepatic TAG and DAG were reduced compared with the HFD group, indicating a net loss of the lipid pool despite an elevation of glycerolipid synthesis, suggesting futile cycling. It is well-known that adipose tissue lipolysis releases more FFAs than is needed for energy production, which requires FFAs to be re-esterified for storage, a process known as FFA/triglyceride cycling^55^. Thus, our observations suggest that in negative energy balance, rapid fat mass breakdown results in excessive release of adipose FFAs that can be buffered by increased hepatic glycerolipid synthesis, which is consistent with the liver being a major site of fatty acid clearance^56^.

An important finding was that when HFD fed mice were switched back to a chow-based diet, they rapidly reduced their energy intake. While energy intake in the HFD fed mice was elevated and explained their weight gain and development of metabolic complications, the diet reversal group reduced their energy intake to levels below that of even the CHOW animals. This was entirely voluntary as the chow was freely available. Interestingly, despite being hypocaloric, the diet reversal mice did not lose muscle mass with the weight reduction being attributed to changes in fat pad mass, indicating these mice were not overtly catabolic. The almost immediate reduction in energy intake upon dietary reversal may be explained by a rapid improvement in leptin sensitivity, as evident by the marked, but not complete reduction in the plasma leptin levels of the reversal mice. Insulin and leptin resistance generally coexist and occur in parallel in response to overfeeding^15^. Thus, given hyperinsulinemia and hence insulin resistance were completely normalized, it is likely leptin resistance was too. However, because the reversal mice were still markedly more obese than the CHOW controls, and the fact that leptin levels are proportional to adiposity, their leptin levels were still considerably higher than the CHOW group. This persistent hyperleptinemia coupled with enhanced leptin sensitivity may explain the reduction in energy intake in the diet reversal mice.

What then, are the mechanisms that could explain the rapid and complete normalization of glucose homeostasis with diet reversal? One possibility relates to improvements in leptin sensitivity. Indeed it has been shown that specifically restoring hypothalamic leptin action in leptin receptor deficient rats improved whole body insulin resistance by enhancing hepatic insulin sensitivity^57^. Thus, enhanced hypothalamic leptin action in the presence of persistently high circulating leptin could exert beneficial effects upon hepatic glucose metabolism, however further studies are required to examine this directly. It is clear that immediately following the diet reversal, the mice voluntarily reduced their total energy intake, again an effect likely mediated via enhanced hypothalamic leptin action. The reduction in energy intake could improve HFD-induced defects in hepatic glucose uptake, which have been well documented to occur in overfeeding or HFD studies in dogs^6^,^7^,^8^,^38^. Given our stable isotope labelled OGTT showed higher hepatic futile glucose cycling, and thus a lower hepatic glucose phosphorylation potential in the HFD fed mice, and the fact that this was completely normalized with the diet reversal, supports this conclusion. Importantly, work in dogs has shown that being in energy surplus by overeating a balanced and nutritious diet is enough to impair hepatic glucose uptake^7^. Unfortunately, it is not possible to directly determine hepatic glucose uptake in mice due to their small size and hence the inability to perform the hepatic portal and hepatic venous catheterization studies required for these measurements. However, the metabolite profiling provides further indirect support that the diet reversal was associated with enhanced hepatic glucose uptake as glycolytic intermediates (ie. G6P, F6P, G3P and PEP) were higher than those measured in the livers from the HFD fed mice.

In conclusion, we show that chronically high-fat high-sucrose fed mice, upon being switched back to a chow-based diet, voluntarily reduce their energy intake resulting in complete normalization of glucose metabolism within 7 days. Interestingly, this was accompanied by a partial reduction in adiposity and hepatic lipid concentrations, indicating that obesity and lipotoxicity *per se* do not necessarily maintain the glucose intolerant and insulin resistant state in HFD fed mice. Rather, persistent over nourishment is likely to be the major factor responsible for causing defects in glucose metabolism. Furthermore, we reveal that during the diet reversal, the liver undergoes a complex metabolic adaptation that involves a reduction in DNL in the face of enhanced fatty acid re-esterification, a mechanism that likely permits net loss of hepatic lipids while preventing an excessive rise in FFA concentrations due to the rapid loss of adiposity.

## Additional Information

**How to cite this article**: Kowalski, G. M. *et al.* Reversing diet-induced metabolic dysregulation by diet switching leads to altered hepatic *de novo* lipogenesis and glycerolipid synthesis. *Sci. Rep.* **6**, 27541; doi: 10.1038/srep27541 (2016).
